# A novel spider toxin as a selective antagonist of the Kv1 subfamily of voltage-gated potassium channels through gating modulation

**DOI:** 10.1016/j.jbc.2025.108341

**Published:** 2025-02-22

**Authors:** Zhen Xiao, Xia You, Minzhi Chen, Huazhen Li, Bo Chen, Zhaotun Hu, Qian You, Hongrong Liu, Piao Zhao, Xi Zhou, Zhonghua Liu

**Affiliations:** 1 The National and Local Joint Engineering Laboratory of Animal Peptide Drug Development, College of Life Sciences, Hunan Normal University, Changsha, China; 2 Peptide and small Molecule Drug R&D Platform, Furong Laboratory, Hunan Normal University, Changsha, China; 3 Institute of Interdisciplinary Studies, Hunan Normal University, Changsha, China; 4 Key Laboratory for Matter Microstructure and Function of Hunan Province, Key Laboratory of Low-dimensional Quantum Structures and Quantum Control, School of Physics and Electronics, Hunan Normal University, Changsha, China; 5 Key Laboratory of Research and Utilization of Ethnomedicinal Plant Resources of Hunan Province, College of Biological and Food Engineering, Huaihua University, Huaihua, China; 6 Institute of Plant Protection, Hunan Academy of Agricultural Sciences, Changsha, China; 7 Hunan Provincial Key Laboratory of Pesticide Biology and Precise Use Technology, Changsha, China

**Keywords:** Kv1 channels, spider toxin, gating modifier toxin, molecular mechanism, gating current

## Abstract

Members of the voltage-gated potassium channel subfamily (Kv1) are essential for the nervous and immune systems, necessitating novel modulators and deeper insights into their structure-function relationships. While all known peptide inhibitors targeting Kv1 channels are pore blockers, we identified MrVIII (κ-HxTx-MrVIII), a novel peptide toxin from the venom of spider *Macrothele raveni*, as the first voltage-gating modifier antagonist with selective activity against Kv1 channels. MrVIII exhibits high-affinity inhibition of Kv1.2, Kv1.3, Kv1.5, and Kv1.6, completely suppressing their currents. By contrast, it selectively inhibits the initial activation phase of Kv1.1, Kv1.4, and Kv1.7 with lower affinity, reflecting its differential subtype modulation. Gating current analyses revealed that MrVIII stabilizes the voltage sensor of Kv1 channels in its resting state, thereby preventing activation upon depolarization. The interaction between MrVIII and Kv1.1, Kv1.4, and Kv1.7 is unstable, with the voltage sensor of Kv1.7, initially stabilized in the resting state by the toxin, potentially transitioning back to an activated state, influenced by the strength and duration of depolarization. Alanine-scanning mutagenesis identified the S3-S4 region as the critical interaction region, with the conserved residue Y339 (in Kv1.3) serving as a key binding site across subtypes. Additionally, the contribution of E283 and T286 in Kv1.1 and A256 in Kv1.7 are key residues in defining channel's pattern in inhibition by MrVIII, compared to Kv1.3. These findings establish MrVIII as a valuable molecular tool for studying Kv1 channels, offering potential pathways for drug development and therapeutic applications.

Voltage-gated potassium channels (Kv channels) conduct K^+^ ion across the cell membrane in response to membrane depolarizations (1). Within this superfamily, the Kv1 (*Shaker*) subfamily, comprising eight members (Kv1.1-Kv1.8), is primarily characterized by activation at subthreshold membrane potentials and slower inactivation kinetics, except for Kv1.4, which exhibits rapid A-type inactivation. The protein sequences of Kv1 channels share over 55% similarity. Structurally, these channels are formed by the assembly of four homomeric or heteromeric subunits, each containing six transmembrane segments (S1–S6). Among these helices, S1–S4 form the voltage-sensing domain (VSD), while S5–S6 constitute the pore domain (2). Kv1 subfamily channels perform diverse biological functions across various tissues and cell types. Among them, the Kv1.3 channel is one of the most extensively studied subtypes. It is highly expressed in effector memory T cells (T_EM_), where it maintains a hyperpolarized membrane potential that facilitates calcium influx, T_EM_ cell activation, proliferation, and cytokine secretion, making it a potential therapeutic target for autoimmune diseases (3, 4). Other subtypes, such as Kv1.1, Kv1.2, Kv1.4, and Kv1.6, are predominantly distributed in the nervous system, with genetic variants linked to severe neurological disorders, including epilepsy, ataxia, and developmental epileptic encephalopathies (5, 6, 7, 8). Furthermore, Kv1.5 is predominantly found in atrial tissue and is associated with atrial fibrillation, while research on Kv1.7 and Kv1.8 remains relatively sparse (9). Regulating Kv1 channel activity holds significant potential as a therapeutic strategy, making pharmacological agents targeting Kv1 channels attractive drug candidates.

Peptide toxins derived from animal venoms have emerged as powerful tools for elucidating ion channel function and provide valuable templates for drug development. These toxins are broadly classified into pore blockers, which physically occlude the ion-conducting pathway, and gating modifiers, which alter voltage sensor movements. A prominent example of a pore blocker targeting the Kv1 subfamily is Shk, a peptide toxin from the sea anemone *Stichodactyla helianthus* (10). Recent cryo-EM structures of Kv1.3 in complex with Shk-186 (Dalazatide) and an antibody-Shk fusion have further elucidated the block mechanism, revealing a critical interaction between Shk-K22 with the backbone carbonyl of Kv1.3-Y447 within the selectivity filter (11, 12, 13). These studies also demonstrated that dalazatide induces structural rearrangements in the selectivity filter, widening it and perturbing ion binding, ultimately locking Kv1.3 in a drug-blocked state. Such findings provide key insights into the molecular basis of toxin-mediated channel inhibition and inform the engineering of therapeutic agents. Dalazatide has successfully completed phase 1b clinical trials in mild-to-moderate plaque psoriasis (14). The high structural similarity among the pore domains of Kv1.3 and other Kv1 subtypes presents significant challenges in achieving subtype selectivity, necessitating extensive engineering efforts, as exemplified by the optimization of Shk into dalazatide (15, 16). Another example is si-544, a peptide derived from *Heterometrus spinnifer* scorpion venom that was optimized for Kv1.3 selectivity through phage display screening and has progressed to clinical trials for atopic dermatitis (ClinicalTrials.gov, NCT05383378) (17, 18). Despite the success of pore blockers in Kv1 channel research and drug development, no gating modifiers targeting Kv1 channels have been identified to date (19). Gating modifiers have been extensively characterized in other ion channel families, including another Kv channel, Nav, and Cav channels, where they often bind to VSD and modulate channel activity (20). Intriguingly, the Kv1.3 S1-S2 loop has been implicated in interactions with endogenous human β-defensin 2; however, its selectivity for other ion channels remains unclear (21). Identifying novel inhibitors and elucidating their mechanism of action could not only advance our understanding of Kv1 channel function but also overcome the limitations posed by current templates and drug-binding site.

In this study, we purified and characterized MrVIII, a novel peptide toxin from the venom of the spider *Macrothele raveni*, as a selective antagonist of the Kv1 subfamily channels and demonstrated its distinct gating modulation effects. Unlike pore blockers, MrVIII selectively interacts with voltage-sensing domains, stabilizing the resting conformation of Kv1 channels. Electrophysiological analyses revealed different inhibition patterns across Kv1 subtypes, with chimeric and mutagenesis studies pinpointing key residues in the S3-S4 region as determinants of subtype selectivity. These findings highlight MrVIII as the first gating modifier for Kv1 channels, providing a valuable molecular tool for ion channel research and drug development.

## Results

### MrVIII is a novel Kv1 channels antagonist

**Figure 1:**
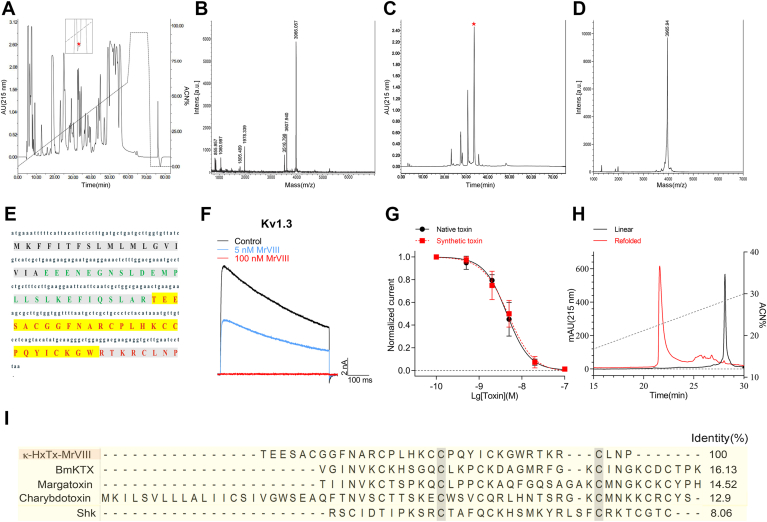
**Purification and characterization of MrVIII.***A*, RP-HPLC profile of spider *Macrothele raveni* venom; the *red asterisk* labeled peak containing MrVIII. *B*, MALDI-TOF MS analysis of the peak containing MrVIII. *C*, ion-exchange HPLC purification of MrVIII to homogeneity; the *red asterisk* labeled peak containing MrVIII. *D*, MALDI-TOF MS analysis of purified MrVIII. *E*, cDNA and protein sequence of MrVIII. The signal peptide, propeptide, and mature peptide were shown in *black*, *green*, and *red bold*, respectively. The sequence determined by Edman degradation was highlighted in *yellow*. *F*, representative current traces showing the Kv1.3 was concentration-dependently inhibited by MrVIII (n = 7). Currents were elicited by a 500 ms depolarization to +30 mV from −80 mV holding. *G*, concentration-response curves of native and synthetic MrVIII inhibiting Kv1.3 currents at +30 mV; the IC_50_ values were determined as 4.4 ± 1.1 nM and 4.8 ± 1.6 nM for native and synthetic toxin, respectively (n = 7). *H*, RP-HPLC purification of crude synthetic (*black*) and refolded (*red*) MrVIII. *I*, sequence alignment of MrVIII with several Kv1 inhibitors from the venomous animals in the database using MEGA8.0. Data are presented as the mean ± SD.

The venom of spider *M. raveni* has remained largely unexplored; we conducted screening the reverse phase high performance liquid chromatography (RP-HPLC) fractions against Kv1.3 transiently expressed in CHO-K1 cells (Fig. 1*A*). This screening analysis confirmed that the fraction marked with a red asterisk exhibited potent inhibition of the Kv1.3 current. Subsequently, we isolated and purified this active fraction using ion-exchange HPLC, and its purity was further verified through matrix-assisted laser-desorption/ionization time-of-flight mass spectrometry (MALDI-TOF MS) analysis (Fig. 1, *C* and *D*). MALDI-TOF MS analysis further revealed that this peak represented a peptide toxin with a molecular weight of 3965.94 Da (M + H^+^). By employing Edman degradation sequencing and analysis of local venom gland cDNA library, we determined the full amino sequence of this toxin and named it MrVIII (rational nomenclature: κ-hexatoxin-MrVIII, κ-HxTx-MrVIII), in accordance with the nomenclature rules proposed by King, G. F (Fig. 1*E*) (22). The theoretical molecular weight of the toxin was 5.7 Da higher than that determined by MALDI-TOF MS analysis, indicating that the six cysteines in its sequence formed three disulfide bonds. This toxin was predicted to contain an inhibitory cystine knot motif based on its cysteine pattern (C_6_-C_19_, C_13_-C_24_, C_18_-C_32_), as modeled by Alphafold 3.0 (23). However, a BLAST search of MrVIII's full sequence in NCBI revealed no significant homology to any toxins or protein, suggesting its unique structure requires experimental validation. Homology analysis with reported Kv1 channel inhibitors indicated that MrVIII shares low similarity with Kv1 channel pore blockers such as BmKTX, margatoxin, and Shk, with a maximum similarity of only 16.1% (Fig. 1*I*) (10, 24, 25, 26). MrVIII concentration-dependently inhibited the peak current of Kv1.3 with the half-maximum inhibitory concentration (IC_50_) of 4.4 ± 1.1 nM at +30 mV (Fig. 1*F* and black curve in Fig. 1*G*). To further confirm the activity of MrVIII, we synthesized linear MrVIII using solid-phase synthesis and reconstructed its disulfide bonds through oxidation refolding (Fig. 1*H*). MALDI-TOF MS analysis confirmed its purity, and its molecular weight was consistent with that of the native toxin. RP-HPLC analysis further demonstrated that the synthetic peptide and native toxin co-eluted as a single peak, indicating their structural similarity (Fig. S1). The concentration-respond curve superimposed with that of the native toxin, with the IC_50_ of 4.8 ± 1.6 nM, which is not significantly different from that of the native toxin (red curve in Fig. 1*G*). These data collectively demonstrated that the synthetic toxin matched the native toxin, thus justifying its use in further experiments.

**Figure 2:**
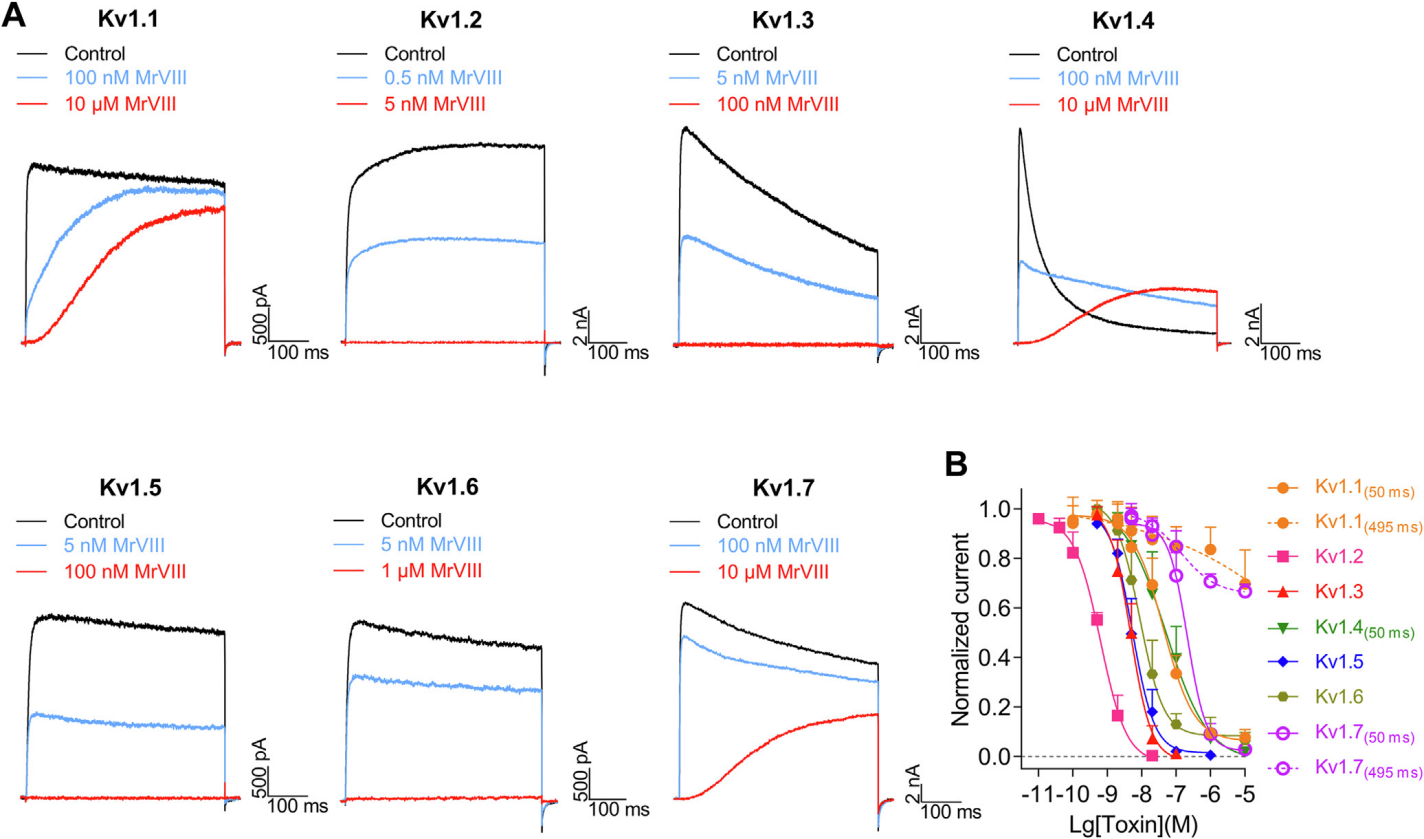
**MrVIII inhibits Kv1 channels with different inhibitory manner.***A*, representative current traces showing the inhibition of Kv1 channels by varying concentrations of MrVIII at a depolarizing voltage of +30 mV (n = 4–7). Holding potential was set −80 mV; currents were elicited by a 500 ms depolarization to +30 mV. *B*, concentration-response curve of MrVIII inhibiting the Kv1 channels. The IC_50_ values for Kv1.2, Kv1.3, Kv1.5, and Kv1.6 were determined as 0.5 ± 0.1 nM, 4.8 ± 1.6 nM, 5.7 ± 2.2 nM, 13.4 ± 6.7 nM, respectively (n = 4–7). For Kv1.1, Kv1.4, and Kv1.7, the currents at 50 ms and 495 ms were measured to evaluate the inhibition. The IC_50(50 ms)_ values in Kv1.1, Kv1.4, Kv1.7 were determined as 50.9 ± 21.7 nM, 62.7 ± 56.9 nM, and 199.0 ± 50.3 nM, respectively. The *dashed curve* was indicated as the response at 495 ms. The inhi%_(495 ms)_ were determined as 23.9 ± 18.5%, 33.3 ± 3.0% for Kv1.1 and Kv1.7, respectively. The 495 ms current of Kv1.4 was enhanced by 3.9-fold in the presence of MrVIII. Data are presented as the mean ± SD.

**Figure 3:**
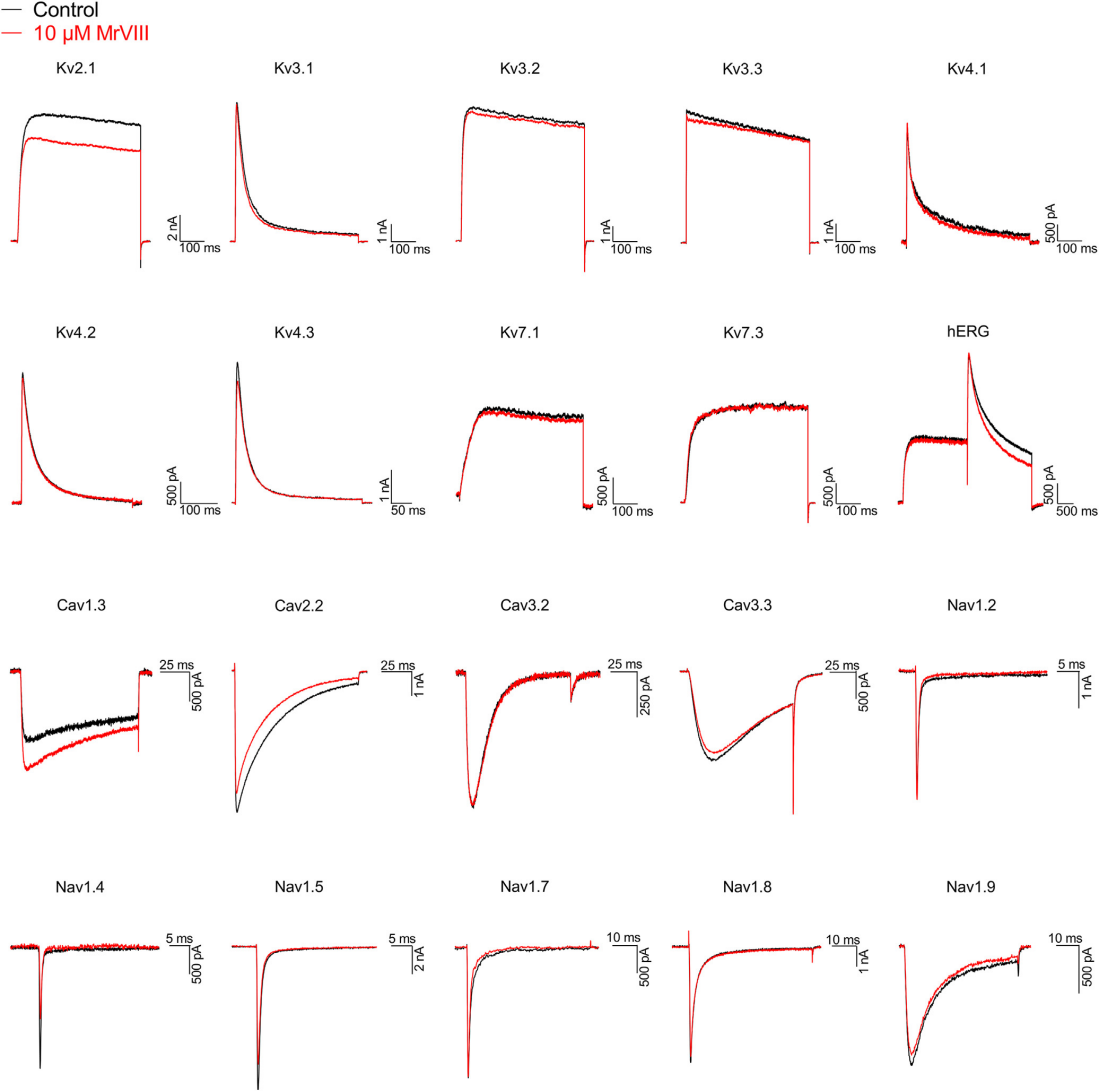
**MrVIII selectively inhibits the Kv1 channel.** At a concentration of 10 μM, MrVIII did not remarkably inhibit currents in non-Kv1 channels. Inhibition ratio were as follows: Kv2.1, Kv3.1-3.3, Kv4.1-4.3, Kv7.1, Kv7.3, hERG, Cav1.3, Cav2.2, Cav3.2-3.3, Nav1.2, Nav1.4-1.5, Nav1.7-1.9 (n = 3–5).

Subsequently, we investigated the activity of MrVIII on other Kv1 subfamily channels. At a depolarizing voltage of +30 mV for Kv1.2, Kv1.5, and Kv1.6, MrVIII fully inhibited their currents with an IC_50_ values of 0.5 ± 0.1 nM, 5.7 ± 2.2 nM, and 13.4 ± 6.7 nM, respectively (Fig. 2, *A* and *B*). However, MrVIII inhibited the initial phase of outward current activation of Kv1.1, Kv1.4, and Kv1.7, which gradually declined during the 500 ms test depolarization. To highlight this intriguing feature, we designated the inhibitory affinity at a time point of 50 ms as IC_50(50 ms)_ and the inhibition ratio at time points at 495 ms by the saturating concentration of 10 μM as inhi%_(495 ms)_. MrVIII inhibited the currents of Kv1.1, Kv1.4, and Kv1.7 with an IC_50(50 ms)_ of 50.9 ± 21.7 nM, 62.7 ± 56.9 nM, and 199.0 ± 50.3 nM, respectively (Fig. 2*B*). At the saturating dose of 10 μM, MrVIII only partially inhibited the current of Kv1.1 and Kv1.7 at 495 ms, with inhi%_(495 ms)_ of 23.9 ± 18.5% and 33.3 ± 3.0%, respectively. Interestingly, the 495 ms current of Kv1.4 was enhanced by 3.9-fold in the presence of MrVIII. Unfortunately, despite extensive efforts, Kv1.8 could not be functionally expressed in our heterologous system, and thus its bioactivity with MrVIII was not tested. In contrast, no remarkable inhibitory effects were observed against several other Kv, Nav, Cav channels even at 10 μM concentration (Fig. 3). Taken toge-ther, these data suggested that MrVIII represents a novel class of peptide toxin capable of the selectively inhibiting Kv1 channels.

### Kinetics of MrVIII interacting Kv1.3

**Figure 4:**
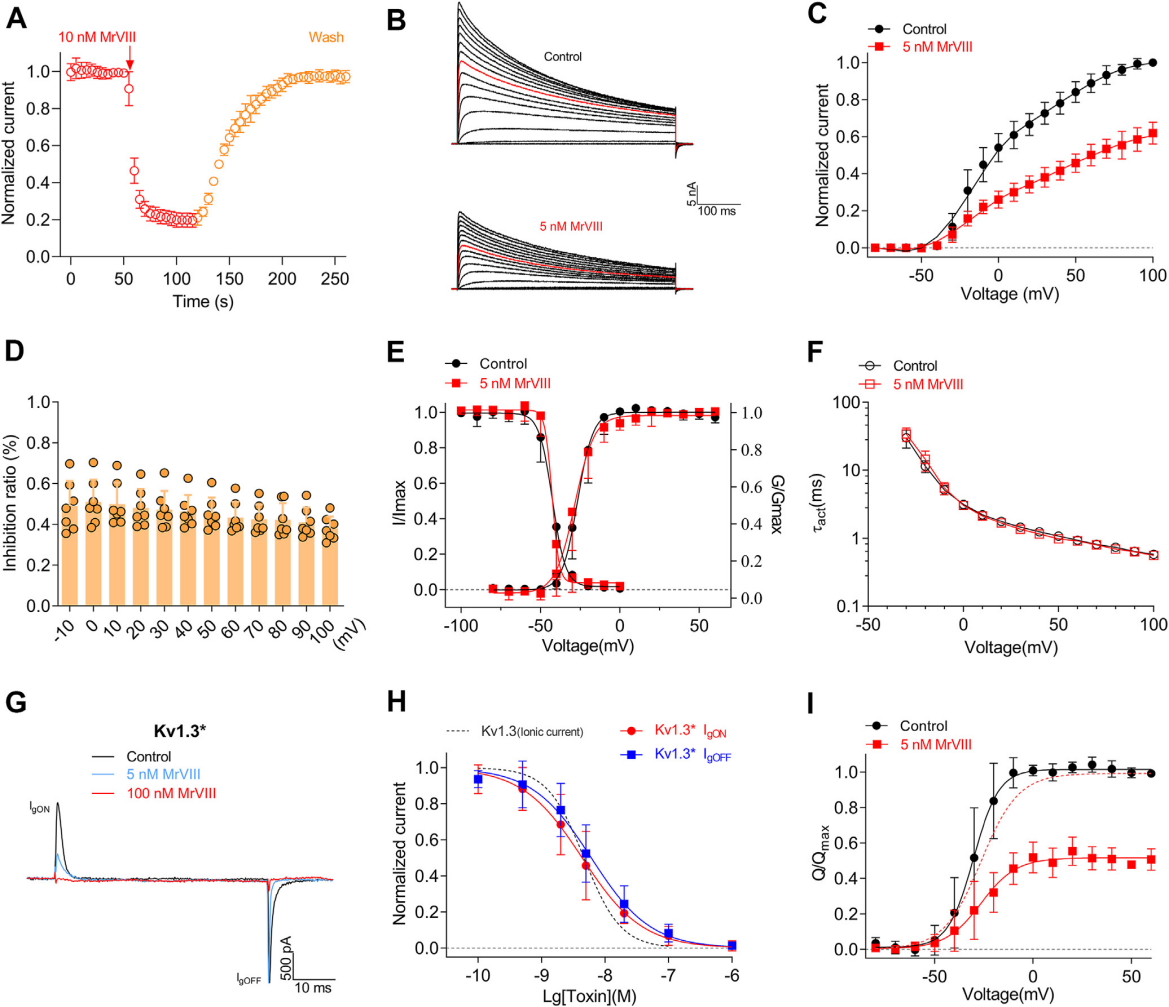
**Kinetics of MrVIII act on Kv1.3.***A*, time course of Kv1.3 current inhibition by 10 nM MrVIII, followed by recovery upon washout with bath solution (n = 6). *B*, representative Kv1.3 currents before (*upper panel*) and after (*lower panel*) 5 nM MrVIII treatment. Currents were elicited by step depolarizations from −80 to +100 mV from a holding potential of −80 mV. The *red* traces show the current at +30 mV (n = 7). *C*, I-V relationships of Kv1.3 before (*black*) and after (*red*) 5 nM MrVIII treatment; current at all voltages were normalized to the control current (before toxin treatment) at +100 mV (n = 7). *D*, the inhibition ratio of 5 nM MrVIII on the currents mediated by Kv1.3 at different depolarizing voltages. No significant difference between groups (ns, *p* ≥ 0.05, one-way ANOVA with *post hoc* analysis using the Dunnett's multiple comparisons test). *E*, the steady-state activation/inactivation curves of Kv1.1 channels before (*black*) and after (*red*) 5 nM MrVIII. *F*, the activation time constants of Kv1.3 before (*black*) and after (*red*) 5 nM MrVIII; the τ_act_ was fitted by a single exponential function (n = 5). No significant difference was observed between the groups (ns, *p* ≥ 0.05, unpaired *t* test). *G*, representative traces showing MrVIII concentration-dependently inhibited the gating current of Kv1.3∗ (Kv1.3/I472C). Cells were held at −100 mV; current elicited by a 50 ms depolarization to +30 mV (n = 8). *H*, concentration-response curve of the I_gON_ (*red*) and I_gOFF_ (*blue*) of Kv1.3∗ inhibited by MrVIII, with an IC_50_ values of 5.0 ± 3.4 nM and 7.0 ± 4.2 nM, respectively (n = 8). Notably, the *black dashed* line representing the concentration-response curve for the ionic current of Kv1.3. *I*, Q_(on)_-V relationships of Kv1.3∗ before (*black*) and after (*red*) 5 nM MrVIII (n = 5). The *red dashed* line representing the Q_(on)_-V relationships of toxin-treated channels by normalizing the Q_(on)_ to their own maximum. Data are presented as the mean ± SD.

To explore the unique mechanism of action, we initially studied how MrVIII inhibits Kv1.3. Due to its importance in drug development, the structure-function relationship and biophysical characteristics of Kv1.3 have been extensively studied. Toxin-binding kinetics assays showed that MrVIII rapidly inhibited Kv1.3 currents, with a time constant of 5.4 ± 1.2 s for toxin association. Dissociation of MrVIII from Kv1.3 was relatively slow, with a time constant of 33.4 ± 8.3 s (Fig. 4*A*). We next analyzed the effect of MrVIII on the current-voltage (I-V) relationships of Kv1.3 (Fig. 4, *B* and *C*). The overall shape of the I-V curve did not shift significantly, and the inhibition ratio at each depolarizing voltage remained consistent, indicating its mode of action lacks voltage dependence (Fig. 4*D*). The conductance-voltage (G-V) curve and steady-state inactivation before and after 5 nM MrVIII nearly overlapped (Control: V_a_ = −26.4 ± 4.6 mV, MrVIII: V_a_ = −27.7 ± 5.1 mV; Control: V_h_ = −42.2 ± 4.7 mV, MrVIII: V_h_ = −42.5 ± 2.4 mV) (Fig. 4*E*). Subsequently, we analyzed the current rise time in response to the depolarized voltage step, which quantified the impact of MrVIII on activation kinetics. In line with the above gating kinetics, MrVIII did not modify the rise time of Kv1.3 at any depolarized voltage; τ_act_ was equivalent to that in control cells (Fig. 4*F*). Thus, at first glance, MrVIII appears to act as pore blocker in a manner similar to previously reported Kv1 channel blockers, but lacking the solid experiment data. Analyzing the regulation of gating current, which arises from the movement of the voltage sensor, is a reliable method to evaluate the modulatory effect of a toxin on the voltage sensor. In this study, we constructed the Kv1.3/I472C mutant (designated as Kv1.3∗) to record gating current, which could accurately analyze I_gOFF_ by eliminating gating charge immobilization (27). Representative current traces in Figure 4*G* demonstrate that MrVIII inhibits both I_gON_ and I_gOFF_ currents in a concentration-dependent manner, with IC_50_ values of 5.0 ± 3.4 nM and 7.0 ± 4.2 nM, respectively (Fig. 4*H*). Notably, there was no significant difference between the IC_50_ values for gating currents and macroscopic ionic currents. Furthermore, 5 nM MrVIII did not alter the voltage sensitivity of the gating machinery, as indicated by the gating charge-voltage (Q-V) relationship. The V_a_ values before and after toxin treatment were −30.1 ± 7.1 mV and −27.4 ± 10.3 mV, respectively, showing no significant difference (Fig. 4*I*). These findings indicate that MrVIII exerts a potent inhibitory effect on the gating currents of Kv1.3 channel, suggesting that the peptide stabilizes the voltage sensor in its resting conformation, thereby inhibiting channel function.

### Differential modulation of MrVIII on Kv1.1 and Kv1.7

**Figure 5:**
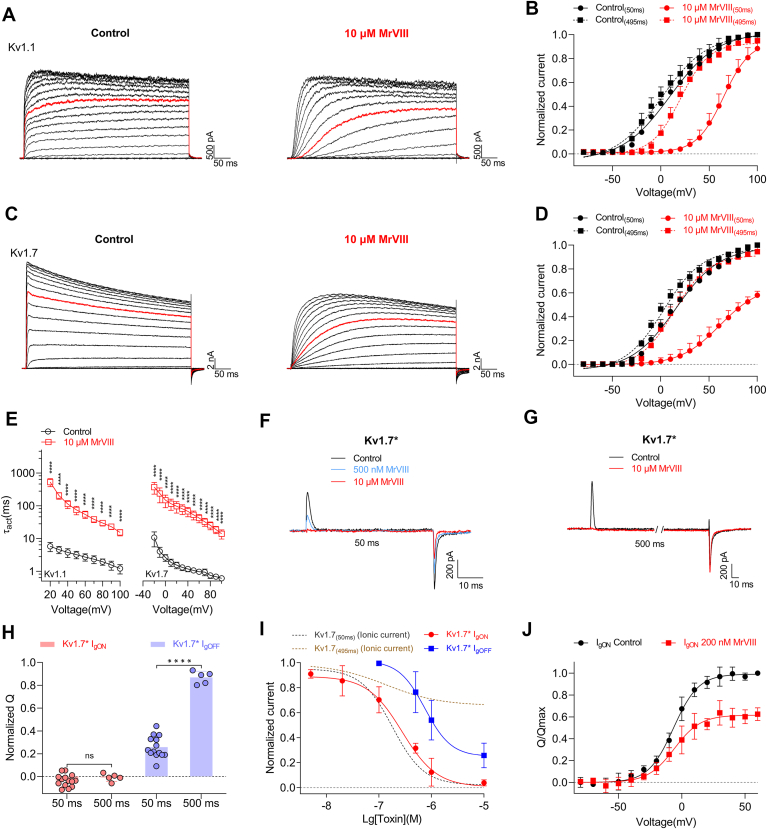
**Differential modulation of MrVIII on Kv1.1, Kv1.7.** Representative Kv1.1 (*A*) and Kv1.7 (*C*) currents recorded before (*left panels*) and after (*right panels*) 10 μM MrVIII treatment. Currents were elicited by step depolarizations from −80 to +100 mV from a holding potential of −80 mV. The *red* traces indicate the current response at +30 mV (n = 5–7). *B* and *D*, I-V relationships of Kv1.1 (*B*) and Kv1.7 (*D*) before (*black*) and after (*red*) treatment with 10 μM MrVIII. In each graph, *solid lines* show I-V curves at 50 ms, and *dashed lines* show I-V curves at 495 ms, highlighting the toxin's impact on channel gating over time (n = 5–7). *E*, the activation time constants of Kv1.1 and Kv1.7 before (*black*) and after (*red*) 10 μM MrVIII; the τ_act_ were fitted by a single exponential function (n = 8–11). Significant difference were observed compared to the toxin-untreated group (∗∗∗∗*p* < 0.0001, paired *t* test). *F*, representative traces showing MrVIII concentration-dependently inhibited the gating current of Kv1.7∗ (Kv1.7/I386C). Cells were held at −100 mV; current elicited by a 50 ms depolarization to +30 mV. Treatment with MrVIII led to a concentration-dependent inhibition of I_gON_ with 10 μM achieving complete inhibition, but the incomplete inhibition of I_gOFF_ was observed (n = 11). *G*, representative gating currents of Kv1.7∗ at a longer depolarization duration (500 ms) before (*black*) and after 10 μM MrVIII treatment (*red*). While 10 μM MrVIII completely inhibited I_gON_, its effect on I_gOFF_ was minimal, indicating a selective inhibitory mechanism that may involve slow dissociation of the toxin from the channel during prolonged depolarization (n = 5). *H*, quantification of the normalized gating charge (Q) for I_gON_ (*red*) and I_gOFF_ (*blue*) at 50 ms and 500 ms depolarization durations, respectively (n = 5–11). No significant difference was observed for I_gON_ (*p* = 0.32) compared to the toxin-untreated group, while a significant difference was observed for I_gOFF_ (∗∗∗∗*p* < 0.0001, unpaired *t* test). *I*, concentration-response curve of the I_gON_ (*red*) and I_gOFF_ (*blue*) of Kv1.7∗ inhibited by MrVIII. The *dashed line* indicated as the concentration-dependent inhibition of MrVIII inhibit the ionic current of Kv1.7 (n = 11). *J*, Q_(on)_-V relationships of Kv1.7∗ before (*black*) and after (*red*) 200 nM MrVIII (n = 5). Data are presented as the mean ± SD.

We explored the molecular mechanism by which MrVIII differentially inhibits Kv1.1 and Kv1.7 while exhibiting distinct effects on Kv1.3. To ensure maximum occupancy, we applied a saturating concentration of 10 μM MrVIII and analyzed the I-V curve. As shown in the representative current traces in Figure 5, *A* and *C*, the current traces following a series of depolarizing voltages exhibit a delayed activation profile, characterized by a gradual ascent to a steady-state plateau in the presence of MrVIII. In the I-V curve of Kv1.1, the inhibition ratio at +50 ms progressively decreased with increasing depolarization, diminishing to only 11.6 ± 7.9% inhibition at +100 mV (Fig. 5*B*). By +495 ms, the inhibitory effect became negligible once the depolarizing voltage exceeded +40 mV, indicating that the inhibition ratio is highly sensitive to the strength of depolarization and may be classified as voltage-dependent inhibition. Consistent with this behavior, MrVIII exhibited an even more pronounced inhibitory effect on Kv1.7. The overall inhibition by MrVIII was stronger than that observed for Kv1.1 at 50 ms, but the currents at 495 ms remain unaffected (Fig. 5*D*). Furthermore, as shown in Figure 5*E*, the rise time of the current was significantly slower in the MrVIII-treated group than the untreated group. Based on the regulatory effect of MrVIII on Kv1.3's voltage sensors and incorporating prior research findings, the following possibilities can be considered: (1) MrVIII may delay the activation of all voltage sensor in Kv1.1 and Kv1.7 upon depolarization, leading to a delayed channel current increase. This is similar to the inhibitory mechanism of spider toxin GxTx on Kv2.1, which delay the upward movement of voltage sensor and alters the G-V curve, resulting in voltage-dependent inhibition (28); (2) under brief depolarization conditions, MrVIII might transiently dissociate from the voltage sensors, allowing the channel to revert to its unbound, activated state, according to the action mechanism of Hanatoxin on Kv2.1/F274A (29).

To explore these possibilities, we focused on the Kv1.7/I386C mutant (Kv1.7∗), which exhibits robust heterologous expression and a strong inhibitory response to MrVIII, enabling detailed analysis of the molecular mechanisms underlying channel inhibition. In contrast, Kv1.1 was not prioritized due to its low expression levels in this system. As shown in Figure 5 *F* and *I*, under stimulation at a depolarizing potential of +30 mV for 50 ms, MrVIII completely inhibited the I_gON_ current of the Kv1.7 channel in a concentration-dependent manner, with an IC_50_ of 214.3 ± 91.8 nM, consistent with its effect on ionic currents. Under 500 nM MrVIII treatment, we subsequently investigated the kinetics of the residual I_gON_ current. The rise time of the residual I_gON_ current was not visibly altered. Furthermore, as shown in Figure 5*J*, MrVIII did not affect the voltage sensitivity of Kv1.7's residual gating charge, with the V_a_ values in the Q-V curve before and after treatment with 500 nM MrVIII being −6.4 ± 2.9 mV and −4.3 ± 3.8 mV, respectively, showing no significant difference. These data confirmed that MrVIII stabilizes Kv1.7's voltage sensors in their deepest resting state, consistent with its action on Kv1.3. Interestingly, as with the decrease in ionic current inhibition ratio over prolonged depolarization, the IC_50_ for I_gOFF_ inhibition could not be determined, as 10 μM MrVIII did not achieve complete inhibition of I_gOFF_. It inhibited only approximately 74.1 ± 9.5% of the I_gOFF_ (Fig. 5*I*). The discrepancy in inhibitory activity on I_gOFF_ may explain its inhibition pattern. Mechanistically, I_gOFF_ originated from the downward movement of the activated voltage sensor, implying that 25.9% of voltage sensors reopen after a 50 ms depolarization at +30 mV. When the depolarization duration was extended to 500 ms, 10 μM MrVIII also completely inhibited the Kv1.7∗ I_gON_. However, its effect on I_gOFF_ was minimal, with the inhibition rate further reduced to only 13.1 ± 5.5% (Fig. 5*H*). These data suggest that MrVIII initially stabilize the voltage sensor in its resting state, but the voltage sensor can still move outward under the driving force of the depolarizing voltage, implying that the stability of channel–toxin complex was disrupted. The lack of I_gON_ signal formation may result from a low signal-to-noise ratio and prolonged depolarization time. Based on these findings, we propose that MrVIII cannot maintain the Kv1.7 voltage sensor in a continuously stable resting state. Depolarization may gradually alter the interaction between the peptide and the voltage sensor, potentially contributing to its unique inhibitory mechanism on Kv1.1 and Kv1.7 channels.

### Variation of S3-S4 in Kv1 channels determines their different modulations by MrVIII

To investigate the structural determinants underlying the differential regulatory effects of MrVIII on Kv1 channels, we employed a chimeric channel strategy. Using Kv1.3 as the template, the S1-S2, S3-S4, and S5-S6 transmembrane helices and their respective linkers from Kv1.1 were inserted to generate chimeras, generating the Kv1.3/1.1 S1-S2, Kv1.3/1.1 S3-S4, and Kv1.3/1.1 S5-S6 chimeric channels. Inhibition assays revealed that MrVIII exhibited IC_50_ values of 1.9 ± 1.1 nM and 3.0 ± 0.9 nM for the Kv1.3/1.1 S1-S2 and Kv1.3/1.1 S5-S6 chimeric channels, respectively, showing similar inhibitory activity on WT Kv1.3 (Fig. S2, *A* and *B*). However, in the Kv1.3/1.1 S3-S4 chimeric channel, MrVIII's inhibitory pattern changed significantly, with an IC_50(50 ms)_ of 230.2 ± 122.8 nM. At 495 ms, the current was enhanced, with inhi%_(495 ms)_ measured as −10.0 ± 13.9%, closely resembling the inhibition pattern observed in WT Kv1.1. These findings indicate that the S3-S4 is the key determinant for the differential inhibition of Kv1.1 and Kv1.3 by MrVIII. To further validate this conclusion, we constructed a reverse chimeric channel using Kv1.1 as the template, inserting the corresponding regions from Kv1.3 into Kv1.1. As shown in Fig. S2, *C* and *D*, experimental results demonstrated that MrVIII completely inhibited the Kv1.1/1.3 S3-S4 current with an IC_50_ of 1.7 ± 0.6 nM. This finding more directly illustrates the exchange of the channel's voltage-dependence phenotype in toxin inhibition by swapping this S3-S4 between Kv1.1 and Kv1.3. Meanwhile, the inhibitory effects of MrVIII on the Kv1.1/1.3 S1-S2 and Kv1.1/1.3 S5-S6 chimeric channels were consistent with those observed for WT Kv1.1, with the IC_50(50 ms)_ values of 103.1 ± 59.1 nM and 48.9 ± 26.6 nM, with inhi%_(495 ms)_ of only 60.5 ± 18.5% and 50.1 ± 19.9%, respectively. In addition to confirming the differential role of S3-S4 between Kv1.1 and Kv1.3, we also substituted the S3-S4 of Kv1.4 and Kv1.7 into Kv1.3 to analyze MrVIII's inhibition profile. The inhibition pattern of Kv1.3/Kv1.4 S3-S4 was significantly altered compared to Kv1.3, with IC_50(50 ms)_ values of 116.6 ± 48.2 nM and an inhi%_(495 ms)_ of only 37.9 ± 5.4%. The IC_50_ for Kv1.3/1.7 S3-S4 could not be fitted, and inhi%_(495 ms)_ was only 23.1 ± 10.0% (Fig. S2, *E* and *F*). Taken together, these data established that the S3-S4 region is the underlying molecular determinant of the differential modulation of Kv1 channels by MrVIII.

### The key determinant residue of Kv1.3 channel in the S3-S4 region inhibited by MrVIII

**Figure 6:**
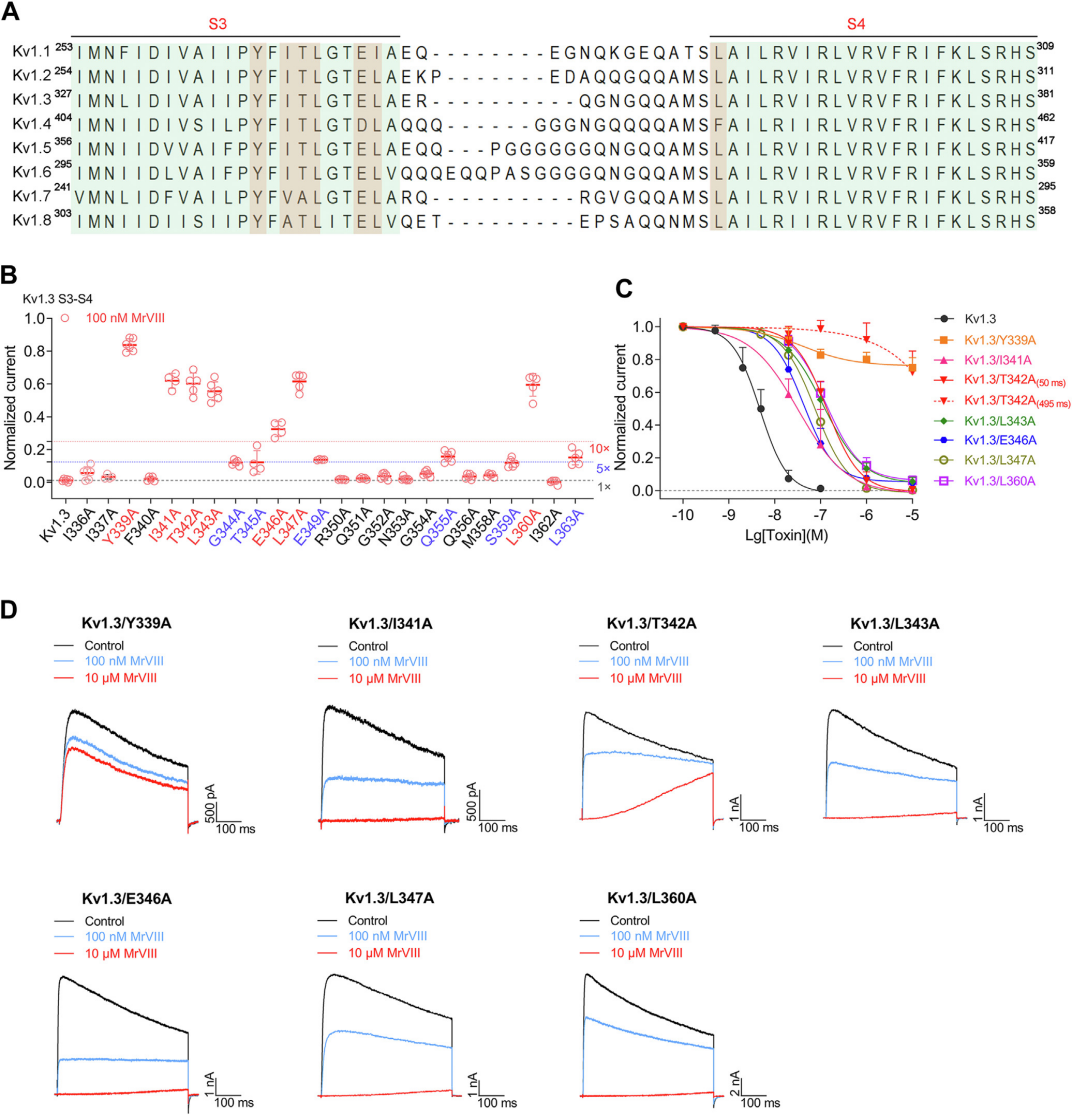
**Identification of key determinant residues in Kv1.3 S3-S4 region critical for MrVIII Inhibition**. *A*, sequence alignment of the S3-S4 region across various Kv1 family channels using MEGA8.0. The transmembrane region of S3 and S4 are highlighted with *cyan* background. Key amino acid residues are highlighted with a *red* background. *B*, summary data of the normalized current response of WT and Kv1.3 mutants at +30 mV after 100 nM MrVIII (n = 4–7). Mutants Y339A, I341A, T342A, L343A, E346A, L347A, and L360A, highlighted in *red* font, exhibit significant reductions in inhibitory potency (more than 10-fold), identifying these residues as critical for MrVIII interaction. Additionally, mutants G344A, T345A, E349A, Q355A, S359A, marked in *blue* font, show notable reductions in inhibitory potency (more than 5-fold), suggesting partial involvement in the binding of MrVIII. *C*, concentration-response curves for MrVIII inhibition of Kv1.3 WT and key alanine mutants (Y339A, I341A, T342A, L343A, E346A, L347A, and L360A) (n = 4–6). Notably, Y339A shows no inhibitory effect, while T342A exhibits poor inhibition at 495 ms, even at 10 μM. *D*, representative current traces for select Kv1.3 alanine mutants (Y339A, I341A, T342A, L343A, E346A, L347A, and L360A) in response to control (*black*), 100 nM MrVIII (*blue*), and 10 μM MrVIII (*red*) conditions (n = 4–6). Currents were elicited by a 500 ms depolarization to +30 mV. Data are presented as the mean ± SD.

MrVIII inhibits Kv1 channels by stabilizing the voltage sensor in its resting state. Structural variations within the S3-S4 region appear to be instrumental in mediating the differential gating modulation exhibited by this peptide across various Kv1 subfamily channels, implying that the S3-S4 may serve as the primary binding site for MrVIII. To investigate this hypothesis, we constructed a series of alanine mutants in the S3-S4 region of the Kv1.3 channel, covering residues I336 to L363, to identify critical interactions between the peptide and the channel (Fig. 6*A*). Among these mutants, the I338A substitution resulted in a loss of functional current, preventing the assessment of MrVIII's inhibitory effect on this particular mutant. For the remaining mutants that retained functional current expression, we evaluated the inhibitory activity of 100 nM MrVIII. As illustrated in Figure 6*B*, the response of these alanine mutants to MrVIII were measured at +30 mV, with the dashed red line representing the inhibitory activity of 10 nM MrVIII against WT Kv1.3 (one-10th of the test concentration). The analysis revealed that MrVIII's inhibitory potency decreased by more than 10-fold for the Y339A, I341A, T342A, L343A, E346A, L347A, and L360A mutants, while the effects on other channel residues were either negligible or minor reductions (up to 5-fold). Further detailed examination yielded the following findings (Fig. 6, *C* and *D*): (i) Kv1.3/Y339A: The inhibitory effect of MrVIII was drastically diminished, with 10 μM MrVIII inhibiting only 24.9 ± 6.0% of the peak current, indicating that Y339 site is a critical binding site for the peptide on the Kv1.3 channel; (ii) Kv1.3/T342A: The inhibition pattern was notably altered, with MrVIII exhibiting an IC_50(50 ms)_ of 142.6 ± 38.2 nM at 50 ms and the inhi%_(495 ms)_ of 27.7 ± 12.9% at 495 ms, suggesting that T342 is essential for stabilizing the interaction between MrVIII and the channel. (iii) Other mutants: The inhibitory activities of MrVIII on other alanine mutants were as follows: Kv1.3/I341A (IC_50_ = 41.0 ± 26.3 nM), Kv1.3/L343A (IC_50_ = 134.9 ± 34.0 nM), Kv1.3/E346A (IC_50_ = 51.1 ± 21.1 nM), Kv1.3/L347A (IC_50_ = 85.7 ± 41.3 nM), and Kv1.3/L360A (IC_50_ = 147.7 ± 60.4 nM). These IC_50_ values, which are approximately 10-fold higher than that of the WT channel, suggest that these residues collectively contribute to the interaction between MrVIII and Kv1.3. The lack of conserved multiple interaction sites may explain the absence of activity on other Kv subfamily channels (Fig. S3). In summary, this study identified key amino acid residues in the S3-S4 region of the Kv1.3 channel that interact with MrVIII, providing a foundation for further exploration into the peptide's mechanism of action.

### Identifying others involved residue of Kv1 channels

**Figure 7:**
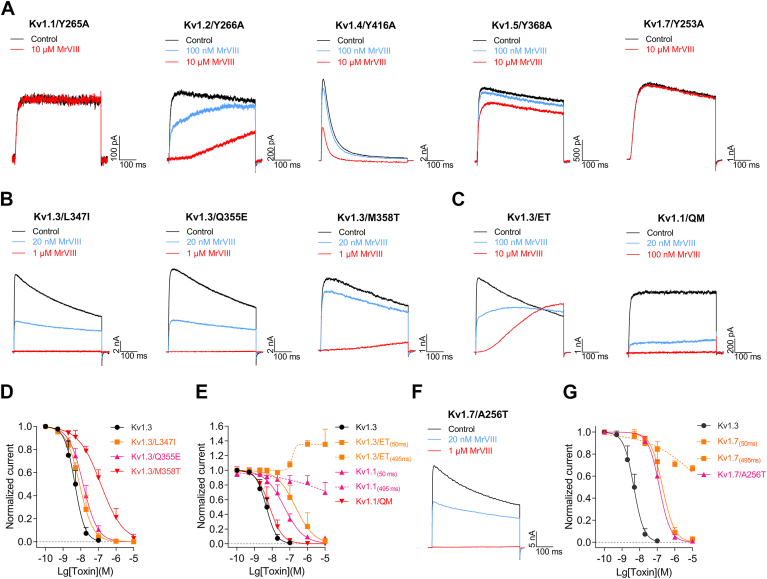
**Characterizing the key residue in the Kv1 channels**. *A*, representative current traces showing Kv1 channel mutans with tyrosine-to-alanine substitutions at position homologous to the Y339 residue in Kv1.3, inhibited by MrVIII (n = 4–6). Currents were elicited by a 500 ms depolarization to +30 mV from −80 mV holding. *B*, representative current traces showing the Kv1.3 and its mutants (L347I, Q355E, M358T) was concentration-dependently inhibited by MrVIII (n = 5). *C*, representative current traces showing MrVIII inhibiting Kv1.3/ET and Kv1.1/QM (n = 3–5). *D*, concentration-response curves of MrVIII inhibiting Kv1.3 WT and Kv1.3 single-point mutants (L347I, Q355E, M358T) (n = 5). *E*, concentration-response curves of MrVIII inhibiting Kv1.3 WT and Kv1.3/ET and Kv1.1/QM mutants, showing a synergistic effect on MrVIII inhibition in the Kv1 channels (n = 3–5). *F* and *G*, representative current trace (*F*) and concentration-response curve (*G*) of MrVIII inhibiting Kv1.7/A256T (n = 6). Data are presented as the mean ± SD.

Building on the identification of key amino acid residues in Kv1.3 channel that interact with MrVIII through alanine-scanning mutagenesis, we extended our analysis to explore these residues across other Kv1 subfamily channels. Notably, Y339 in Kv1.3 was identified as a critical determinant for peptide binding. Accordingly, alanine mutations were introduced at homologous positions in other Kv1 channels, except for Kv1.6/Y307A, which failed to express functional currents. As depicted in Figure 7*A*, MrVIII exhibited diminished inhibition of the homologous mutants in Kv1.1, Kv1.4, Kv1.5, and Kv1.7 channels. Specifically, 10 μM MrVIII inhibited Kv1.1/Y256A, Kv1.4/Y416A, Kv1.5/Y368A, and Kv1.7/Y253A by −9.5 ± 4.4%, 63.5 ± 18.4%, 19.1 ± 6.2%, and −9.4 ± 13.6%, respectively. Notably, despite high concentrations, MrVIII did not slow down the inactivation of Kv1.4/Y416A, further highlighting the conserved tyrosine's role in peptide binding across Kv1.1, Kv1.3, Kv1.4, Kv1.5, and Kv1.7 channels. However, MrVIII retained substantial inhibitory potency on Kv1.2/Y266A mutant, albeit without fully blocking the channel current. The IC_50(50 ms)_ was determined to be 418.5 ± 41.2 nM at 50 ms and the inhi%_(495 ms)_ of 36.9 ± 14.1% at 495 ms. These data suggest that MrVIII exerts a different inhibitory mechanism on Kv1.2 relative to other Kv1 channels. The profound reduction in MrVIII's effect across homologous mutants supports the idea that it binds to a conserved region.

Sequence alignment of the Kv1 subfamily revealed that the divergence between Kv1.3 and Kv1.1 channels is localized within the extracellular loop of the S3-S4 region, particularly spanning residues 350 to 353 in Kv1.3. However, prior alanine-scanning results indicated that these fragments do not significantly contribute to peptide–channel interaction. In contrast, differences at positions 347, 355, and 358 between Kv1.3 and Kv1.1 prompted further analysis. To explore whether these discrepancies influence peptide-mediated inhibition, we substituted these residues in Kv1.3 with their homologous counterparts in Kv1.1 and assessed the MrVIII's inhibitory efficacy on these single-point mutants. Under a +30 mV stimulus, the IC_50_ values for Kv1.3/L347I, Kv1.3/Q355E, and Kv1.3/M358T mutants were 14.2 ± 0.8 nM, 14.2 ± 7.2 nM, and 142.8 ± 33.0 nM, respectively (Fig. 7, *B* and *D*). Interestingly, single-point mutations alone were insufficient to reverse the differential inhibitory profiles observed between Kv1.3 and Kv1.1. To further investigate this, we introduced double adjacent mutations around Q355 and M358 in Kv1.3 to explore potential synergistic effects on MrVIII's inhibitory activity. Experimental results showed that MrVIII's inhibition profile on the Kv1.3/ET (Kv1.3/Q355 E_M358T) mutant was significantly altered, with an IC_50(50 ms)_ of 122.3 ± 24.5 nM and the inhi%_(495 ms)_ of −35.4 ± 20.7% at 495 ms, closely mirrored its activity on Kv1.1 (Fig. 7, *C* and *E*). Conversely, MrVIII fully inhibited the Kv1.1/QM (Kv1.1/E283Q_T286M) mutant, with an IC_50_ of 7.7 ± 5.3 nM, aligning with its effect on Kv1.3 (Fig. 7, *D* and *E*). These results demonstrate that Q355 and M358 in Kv1.3, E283, and T286 in Kv1.1 worked synergistically in defining channel's inhibition phenotype by MrVIII.

In our previous alanine-scanning study on the Kv1.3 channel, we identified that the Kv1.3/T342A mutant exhibited a slow phase of activation. This observation prompted further exploration of the potential function of the homologous residue in the Kv1.7 channel, where the corresponding site is occupied by an alanine. Experimental data revealed that MrVIII fully inhibited the Kv1.7/A256T mutant current at +30 mV, with an IC_50_ of 138.0 ± 50.3 nM, showing no decrease in inhibitory effect upon depolarization (Fig. 7, *F* and *G*). However, despite the relatively strong inhibition of the Kv1.7/A256T mutant, its potency was approximately 28-fold weaker than that of the Kv1.3 channel. These findings suggest that, in addition to the A256 residue, other regions or residues in the Kv1.7 channel may contribute to peptide interaction, likely through cooperative mechanisms, ultimately modulating MrVIII's inhibitory activity.

## Discussion

Kv channels are essential for maintaining cellular membrane potential, regulating excitability, and facilitating signal transduction. Among them, the Kv1 subfamily plays a crucial role in the central nervous system and immune responses, making the exploration of its structure-function relationships and modulators highly significant. Peptide toxins, known for their high specificity and efficiency, have become indispensable in ion channel research. In this context, MrVIII, a novel peptide toxin from spider *M. raveni*, emerges as an exciting discovery. Unlike classical pore blockers, MrVIII acts as a voltage-gating modifier, interacting with the VSD to stabilize Kv1 channels in their resting state, with high affinity and selectivity.

Voltage-gating modifiers stabilize the VSD in a resting conformation, preventing activation in response to depolarizing voltages. This interaction increases the energy barrier required for channel opening, resulting in a depolarizing shift in the I-V and G-V curves. Typically, this results in voltage-dependent inhibition, where strong depolarization can partially or fully counteract the inhibitory effect on voltage sensor (20, 29). However, MrVIII presents a distinct case, as it does not significantly alter the steady-state activation or inactivation properties of Kv1.3 channels. Its classification as a gating modifier has been confirmed through direct inhibition of gating currents. This unusual inhibitory profile likely stems from a strong and stable binding interaction between the peptide and the channel, resisting dissociation even under depolarizing conditions—an interaction mechanism that resembles that of HWTX-IV with Nav1.7 channels (30). Such stability has been crucial in studies of ion channel–toxin complexes, as evidenced by structural analyses of HWTX-IV in complex with Nav1.7 and the chimeric NaChBac channel containing the domain II S3-S4 loop of Nav1.7 (31, 32). In addition, unlike other Kv subfamilies where key residues for gating modifier toxin binding are primarily located in the C-terminal half of the S3 segment, the critical residues for MrVIII's interaction with Kv1.3 are situated at both ends of the S3-S4 loop, with Y339 in the S3b region playing a pivotal role. Alanine-scanning mutagenesis confirmed the importance of the S3-S4 of the VSD in MrVIII binding, underscoring the critical interaction pattern of MrVIII with Kv1 channels. Interestingly, while MrVIII inhibits Kv1.2, Kv1.5, and Kv1.6 channels similarly to Kv1.3, its inhibitory effect on Kv1.2 appears to differ slightly. Specifically, MrVIII demonstrates stronger inhibitory activity on Kv1.2, and there is a lack of dramatic reduction in current amplitude in the Kv1.2/Y266A mutant, suggesting a possible variation in the binding mechanism or channel conformation. The precise details of this difference remain unclear and warrant further investigation. Unfortunately, molecular docking analysis of the interaction between MrVIII and Kv1.3 was not achievable in this study, primarily due to the depolarized state of the available Kv1.3 structures and the limited resolution of its extracellular interaction surface. Despite this limitation, our findings firmly establish MrVIII as a voltage-gating modifier, providing a valuable molecular tool for dissecting the gating mechanisms of Kv1 channels.

MrVIII selectively inhibits the initial activation phase of Kv1.1, Kv1.4, and Kv1.7, a pattern reminiscent of the inhibitory effects of Hanatoxin on the Kv2.1/F274A mutant and the *Shaker* channel (29, 33). In the Kv2.1/F274A mutant, Hanatoxin dissociation contributes to the inhibitory pattern, with the toxin dissociating from its target channel over hundreds of milliseconds, unlike the rapid modulation observed with GxTx on Kv2.1, where fluorescently labeled toxin binds and dissociates upon depolarization (34). However, for the *Shaker* channel, Hanatoxin is thought to remain bound, indicating that the precise mechanism of this inhibition patter remains elusive. In case of MrVIII, both the slower movement of the toxin-bound voltage sensor and potential dissociation could contribute to its effects on Kv1.7, with these mechanisms warranting further examination. The stability of the interaction network between MrVIII and the VSD appears to govern the toxin's distinct inhibitory effect. This network is finely tuned by multiple binding sites, and disruption of these interactions, such as the alanine mutation of T342 in Kv1.3, weakens MrVIII's inhibitory potency and restores the voltage-dependence phenotype typical of Kv1.7. These findings underscore the crucial role of specific residues within the S3-S4 region in maintaining the stability of the toxin–channel interaction. Moreover, the interaction between MrVIII and Kv1.1 or Kv1.7 appears to be synergistically regulated by multiple binding sites within the S3-S4 region, contributing to the formation of a relatively unstable complex. This synergistic modulation likely explains the differential inhibitory patterns observed across these channels. These observations align with the varying degrees of voltage-dependent inhibition exhibited by other peptide toxins, such as Hanatoxin and κ-LhTx-1, where subtle structural changes in the paddle motif substantially influence gating modulation (35). In Kv1.4, MrVIII interferes with N-type inactivation, likely by disrupting the allosteric coupling between N-terminal inactivation ball and gating mechanism (36). This unique effect highlights MrVIII's potential as a molecular tool to further dissecting the inactivation kinetics of Kv1.4.

In conclusion, MrVIII emerges as a potent voltage-gating modifier of Kv1 subfamily channels, offering both a valuable molecular tool for studying channel gating and a promising template for the designing subtype-selective inhibitors. Its distinctive interaction mechanism, characterized by multisite binding along the S3-S4 region, provides insightful understanding of the modulation of channel activity by peptide toxins. This multisite interaction strategy underscores the importance of considering multiple binding sites in inhibitor design to achieve both specificity and efficacy. Moreover, the voltage-dependent inhibition exhibited by MrVIII on Kv1.1, Kv1.4, and Kv1.7, rather than complete blockade, presents a potential therapeutic advantage. For example, in the clinical treatment for *KCNA2* gain-of-function variants, partial inhibition may mitigate severe off-target effects or toxicity associated with complete channel suppression (37). These efforts will not only deepen our understanding of Kv1 channel gating modulation but also facilitate rational drug design of safe and selective Kv1 modulators with clinical applications.

## Experimental procedures

### Venom and toxin purification

Spider *M. raveni* were collected from Guangxi Province. The spiders, with body length ranging from 3 to 5 cm, were subjected to electric stimulation to extract venom. The collected crude venom was lyophilized and stored at −80 °C until further use. The crude venom was dissolved in ddH_2_O to a final concentration of 5 mg/ml and subjected to the first round of RP-HPLC purification (acetonitrile gradient: 0%–60%, at an increasing rate of 1% per minute). The fraction containing MrVIII was collected, lyophilized, and further purified using anion ion-change purification. The purity and molecular weight of the toxin were tested by MALDI-TOF MS analysis.

### Toxin characterization

The N-terminal sequence of MrVIII, comprising 27 amino acids, was determined *via* Edman degradation using an automatic protein sequencer (SHIMADZU PPSQ31A, Kyoto). The full sequence was subsequently identified by blasting the N-terminal sequence against the local protein sequences database derived from the venom gland cDNA library of *M. raveni* (unpublished data).

### Solid-phase peptide synthesis

MrVIII was synthesized manually by using the Fmoc (N-(9-fluorenyl) methoxycarbonyl/tert-butyl strategy and 1-hydroxybenzotrazole/2-(1H-benzotriazole-1yl)-1,1,3,3-tetramethyluronium tetrafluoroborate/N-methylmorpholine coupling method. The desired pooled fractions containing the linear reduced peptide were concentrated and lyophilized before folding. The linear peptide was dissolved in ddH_2_O and diluted into the refolding buffer composed of 0.1 M NaCl, 5 mM GSH, 0.5 mM GSSG, and 0.1 M Tris–HCl (pH 7.4, adjusted with HCl). After stirring the solution at 4 °C for 12 h, the reaction was terminated by adding TFA to a final concentration of 0.1%, followed by RP-HPLC purification to collect the correctly refolded toxin. The molecular weights of the linear and refolded peptides were confirmed *via* MALDI-TOF MS analysis.

### Plasmids and site-directed mutation

The cDNA of hKv1.1, hKv1.2, hKv1.3, rKv1.4, hKv1.5, mKv1.6, and hKv1.7 were subcloned into mammalian expression vectors pcDNA3.1 or pCMV-blank vectors, as well as other plasmids described previously (35). Channel mutants were generated by site-directed mutations. Briefly, the parental channel plasmid was amplified using a pair of oppositely directed primers harboring the designed mutation site, with KOD Fx (TOYOBO Co, Ltd). The resulting PCR mix was treated with FastDigest DpnI (Thermo Fisher Scientific) to remove the methylated template. Ten microliters of the digested product was then used to transform 100 μl *E. coli* DH5α competent cells. Transformants were randomly picked and sequenced to confirm the presence of the intended mutation.

### Cell culture and transient transfection

CHO-K1 cells were selected for expressing Kv channels due to their lack of endogenous Kv channels, while HEK293 or ND7/23 cells were used for Nav and Cav channel expression. CHO-K1 cells were cultured in a Dulbecco's modified Eagle's medium/Ham's F-12 nutrient mixture (DMEM-F12) medium, and HEK293T and ND7/23 cells in DMEM medium, all supplemented with 10% fetal bovine serum and 1% penicillin-streptomycin (all from Gibco, Thermo Fisher Scientific, Inc) under standard conditions (37 °C, 5% CO_2_, and saturated humidity). These cell lines were purchased from Stem Cell Bank, Chinese Academy of Sciences. Channel plasmids were cotransfected with pEGFP-N1 (encoding GFP) into the respective cells using lipofectamine 2000, following the manufacturer's instructions (Invitrogen, Thermo Fisher Scientific, Inc.). Nav1.9-GFP plasmid was transfected into ND7/23 using the X-tremeGENE HP DNA Transfection Reagent (Roche). After 4 to 6 h transfection, cells were seeded onto poly-lysine–coated coverslips, and 24 to 36 h later, transfected cells were ready for patch-clamp analysis.

### Electrophysiology

Patch clamp experiments were conducted using an electrophysiology platform comprising a MultiClamp 700B amplifier and an Axon Digidata 1550 AD/DA convertor (Axon Instruments). Data acquisition was performed with pClamp 10.5 software (Axon Instruments). All experiments were performed at room temperature.

### Whole-cell ionic current recordings

For Kv current recordings, the bath solution contained (in mM) the following: 140 NaCl, 5 KCl, 1 MgCl_2_, 2 CaCl_2_, 10 glucose, and 10 Hepes (pH 7.3, adjusted with NaOH), and the pipette solution contained (in mM) the following: 140 KCl, 2.5 MgCl_2_, 11 EGTA, and 10 Hepes (pH 7.3, adjusted with KOH). Series resistance was maintained below 10 MΩ and compensated to 80%. Concentration–response curves were fitted by the Hill equation to determine the toxin's potency. Whole cell conductance (G) at each depolarizing voltage (V) was determined using the equation: G = I/(V−V_rev_), where I is the current amplitude, and V_rev_ is the reversal potential. The reversal potential for K^+^ current was estimated to be −85.6 mV using the Nernst equation. G-V curves were obtained by plotting the normalized conductance as a function of V and fitted by the Boltzmann equation: y = 1/(1+ exp((V_a_-V)/k)), which V_a_ is the midpoint voltage, V is the test potential, and k is the slope factor. The steady-state fast inactivation of Kv1.3 was measured using a two-pulses protocol. The cell was held at −120 mV, and a train of conditional voltages (−120–40 mV in 10 mV increments, 60 s) were applied to induce channel inactivation, followed by a +60 mV test pulse (300 ms) to assess the proportion of noninactivated channels. The sweep interval was set to 30 s. Currents at the test pulse (I) were normalized to the maximum value (I_max_) and plotted as a function of the conditional voltage (V). The curve was fitted by the Boltzmann equation: I/I_max_ = A+(1 − A)/(1+exp ((V−V_h_)/K)), where V_h_ is the half-maximum inactivation voltage, A represents the minimum channel availability, and k is the slope factor.

### Gating-current recordings

Gating currents were recorded by transfecting HEK293T cells with a saturating dose of plasmid to ensure maximal channel expression. The Kv1.3/I472C and Kv1.7/I386C mutants were used to record the I_gOFF_, minimizing slow voltage sensor return as reported in previous studies (28). The bath solution contained (in mM) the following: 140 NMDG, 50 Hepes, 2 CaCl_2_, 2 MgCl_2_, 0.1 EDTA, and 0.01 CsCl (pH 7.3, adjusted with methanesulfonic acid), and the pipette solution contained (in mM) the following: 140 NMDG, 1 NMDG-Cl, 50 HF, 5 EGTA, and 50 Hepes (pH 7.3, adjusted with methanesulfonic acid). Gating charge movement (Q) was determined by integrating the area under the I_gON_ and I_gOFF_ traces, representing voltage sensor movement in response to depolarizing pulses. The data were normalized and plotted against voltage to generate Q-V curves, which were fitted using a Boltzmann function to estimate the voltage dependence of charge movement.

### Data analysis

Data were presented as mean ± SD, with n denoting the number of independent cells analyzed. Data analysis was performed using pClamp 10.5 software (Axon Instruments), GraphPad 9.5.1 (GraphPad Software), and Excel 2021 (Microsoft Corporation). Statistical significance was assessed by two-tailed *t* test or one-way ANOVA. Significance thresholds in the figure panel were represented as follows: ns. *p* ≥ 0.05, ∗*p* < 0.05, ∗∗*p* < 0.01, ∗∗∗*p* < 0.001, and ∗∗∗∗*p* < 0.0001.

## Data availability

Data is presented in the manuscript.

## Supporting information

This article contains supporting information Figs. S1–S3.

## Conflict of interest

The authors declare that they have no conflicts of interest with the contents of this article.
