# Supplementary material for: A novel spider toxin as a selective antagonist of the Kv1 subfamily of voltage-gated potassium channels through gating modulation
Source: J Biol Chem. 2025 Feb 22;301(4):108341. doi: 10.1016/j.jbc.2025.108341 (PMC11984586; doi:10.1016/j.jbc.2025.108341)

**Supporting information**

**Supplementary figure 1.** RP-HPLC co-elution experiment of native and synthetic MrVIII

**Supplementary figure 2**. Differential inhibition of Kv1 channel chimeras by MrVIII reveals the key role of the S3-S4 region in modulation specificity

*A*, Representative currents showing MrVIII concentration-dependently inhibited Kv1.3/1.1 chimeric channels with S1-S2, S3-S4, or S5-S6 from Kv1.1 inserted into Kv1.3 (n = 4-5). Currents were elicited by a 500 ms depolarization to +30 mV from a holding potential of -80 mV. *B*, Concentration-response curves for MrVIII inhibition of Kv1.3 and Kv1.3/Kv1.1 chimeric channels, showing that Kv1.3/1.1 S3-S4 exhibited an altered inhibitory pattern resembling Kv1.1 (n = 4-5). *C*, Representative current traces of Kv1.1/1.3 chimeric channels with S1-S2, S3-S4, or S5-S6 regions from Kv1.3 inserted into Kv1.1 (n = 4-7). *D*, Concentration-response curves for MrVIII inhibition of Kv1.1 and Kv1.1/1.3 chimeric channels (n = 4-5). *E*, Representative current traces of Kv1.3/1.4 S3-S4 and Kv1.3/1.7 S3-S4 chimeric channels, in the absence (black) or presence of MrVIII at 100 nM (blue) and 10 μM (red) (n = 5-6). *F*, Concentration-response curves for MrVIII inhibition of Kv1.3/1.4 S3-S4 and Kv1.3/1.7 S3-S4 (n = 5-6). Data are presented as the MEAN ± SD.

**Supplementary figure 3.** Sequence alignment of key interaction sites of Kv1.3 with MrVIII and other Kv subfamily channels.

Amino acid residues exhibiting a reduction in IC_50_ by more than 10-fold are highlighted in red, while those showing a reduction of more than 5-fold are marked in blue. The alignment was performed using MEGA8.0.

**Supplementary figure 1**

**
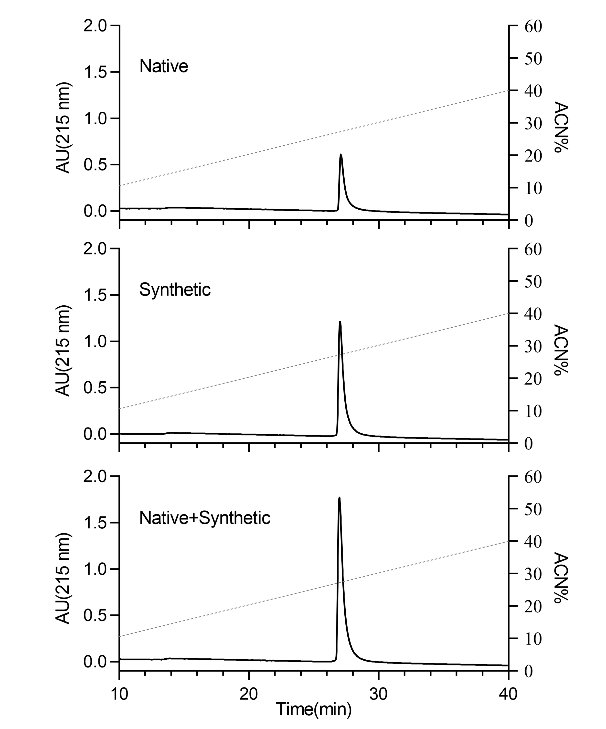
**


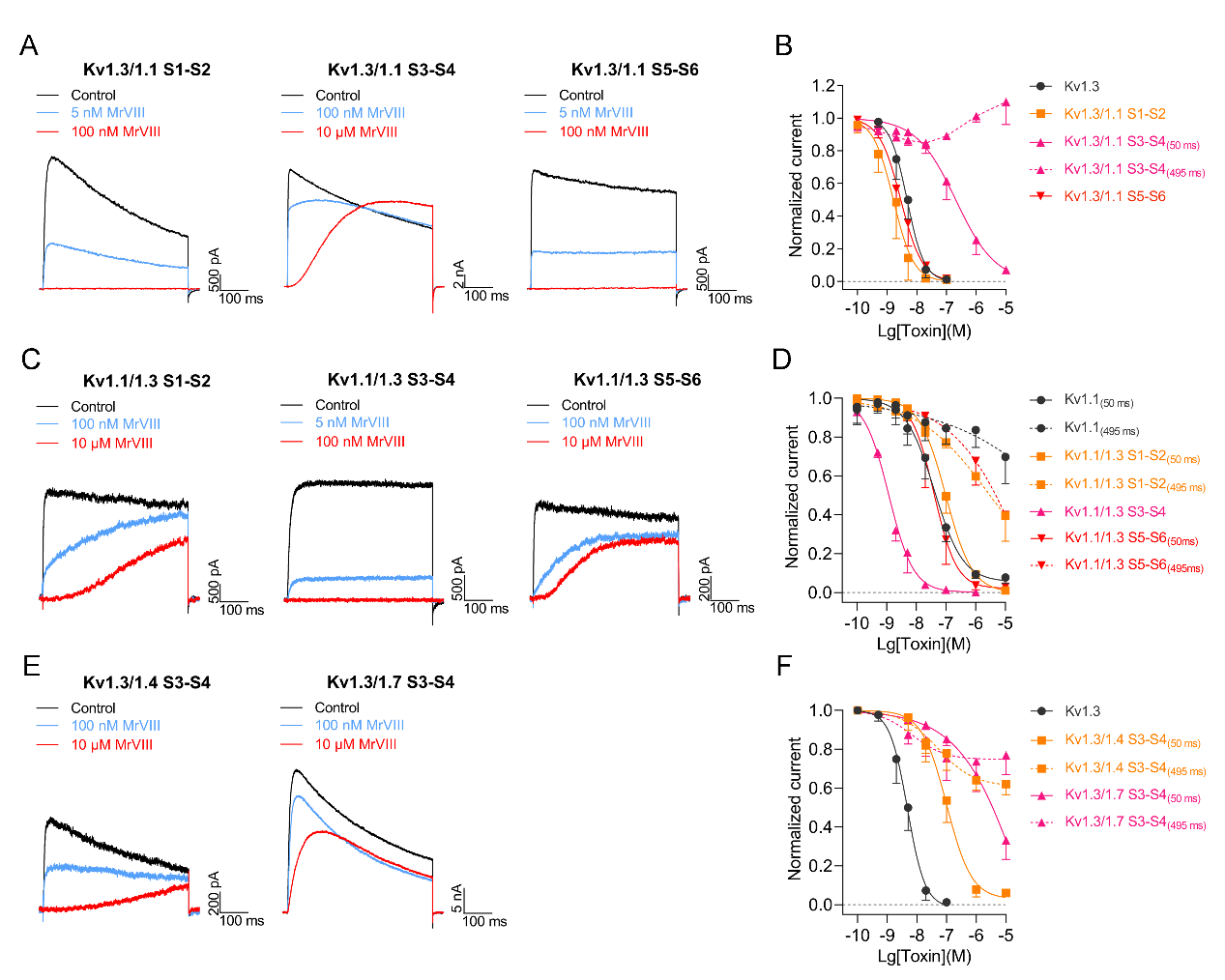
**Supplementary figure 2**

**Supplementary figure 3**


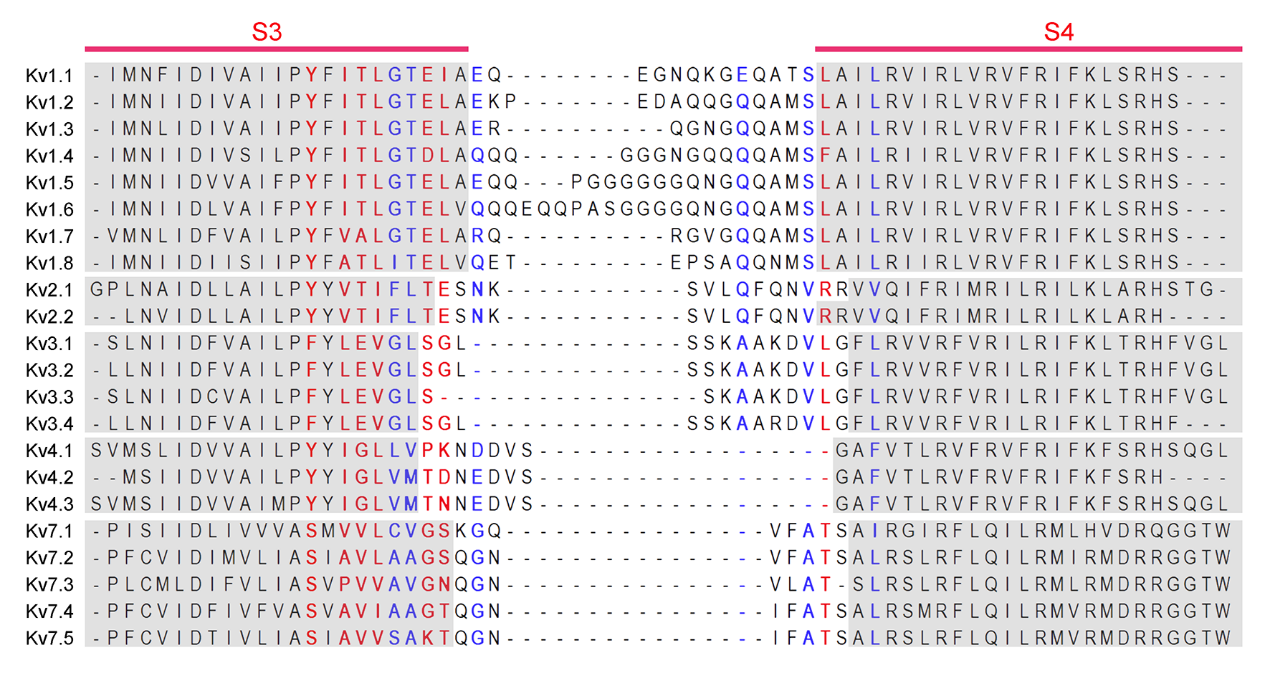

Supplement: Supporting information [file mmc1.docx]
